# Genetic analysis of porcine circovirus type 2 from pigs affected with PMWS in Chile reveals intergenotypic recombination

**DOI:** 10.1186/s12985-017-0850-1

**Published:** 2017-10-04

**Authors:** Victor Neira, Natalia Ramos, Rodrigo Tapia, Juan Arbiza, Andrónico Neira-Carrillo, Manuel Quezada, Álvaro Ruiz, Sergio A. Bucarey

**Affiliations:** 1 Departamento de Medicina Preventiva, Facultad de Ciencias Veterinarias y Pecuarias, Universidad de Chile, Santiago, Chile; 2 Sección Virología, Facultad de Ciencias, Universidad de la República, Montevideo, Uruguay; 3 Laboratorio Polyforms, Departamento de Ciencias Biológicas Animales, Facultad de Ciencias Veterinarias y Pecuarias, Universidad de Chile, Santiago, Chile; 4 Facultad de Ciencias Veterinarias, Universidad de Concepción, Chillán, Chile; 5 Centro Biotecnológico Biovetec, Departamento de Ciencias Biológicas Animales, Facultad de Ciencias Veterinarias y Pecuarias, Universidad de Chile, Santiago, Chile

**Keywords:** PCV2, PMWS, Genetic diversity, Recombination

## Abstract

**Background:**

Porcine circovirus type 2 (PCV2) is a very small, non-enveloped and icosahedral virus, with circular single stranded DNA genome. This virus is the most ubiquitous and persistent pathogen currently affecting the swine industry worldwide. PCV2 has been implicated as the major causative agent of postweaning multisystemic wasting syndrome (PMWS), a disease which is characterized by severe immunosuppressive effects in the porcine host. Worldwide PCV2 isolates have been classified into four different genotypes, PCV2a, PCV2b, PCV2c and PCVd. The goal of this work was to conduct the first phylogenetic analysis of PCV2 in Chile.

**Methods:**

PCV2 partial ORF2 sequences (462 nt) obtained from 29 clinical cases of PMWS in 22 Chilean intensive swine farms, covering over the 90% of the local pork-production, were analyzed.

**Results:**

14% and 52% of sequences belonged to the genotypes PCV2a and PCV2b, respectively. Surprisingly, 34% of sequences were PCV2a/PCV2d recombinant viruses.

**Conclusions:**

Our findings suggested that a novel cluster of Chilean sequences emerged resulting from intergenotypic recombination between PCV2a and PCV2d**.**

## Background

*Porcine Circovirus type 2* (PCV2) is a small non-enveloped virus, member of the genus *Circovirus*, family *Circoviridae* [1]. PCV2 is primary causative agent of postweaning multisystemic wasting syndrome (PMWS) and other diseases in swine referred to as porcine circovirus associated diseases (PCVAD) [2]. PMWS was first described in Canada in 1991, and nowadays it has been described in pig population worldwide, having a serious economic impact on the global swine industry. Pigs affected with PMWS show decreased weight gain, progressive weight loss, enlarged lymph nodes, and others non-specific clinical signs such as dyspnea and diarrhea [3] .

The genome of PCV2 is a 1.76–1.77 kb single-stranded circular DNA, containing three main open reading frames (ORFs) which encode viral proteins [4]. ORF1 encodes for the replicase protein (Rep) and splice variants involved in viral replication, ORF2 encodes for the capsid (Cap), an exclusive structural protein [5], and ORF3 encodes a non-structural protein which causes apoptosis in PCV2 infected cells and has been associated with pathogenicity [6]. PCV2 isolates could be classified into four different genotypes, PCV2a, PCV2b, PCV2c and PCV2d, based on pairwise sequence comparisons (PASC) [7], and most recently based on reference sequences and/or marker positions [8]. PCV2a and PCV2b are the major groups, worldwide distributed, while PCV2c has been described only in Denmark from swine historic archived tissues [9] and recently from wild boar in Brazil [10]. PCV2d correspond to a PCV2b mutant (mPCV2b) described in some countries of Europe, Asia, North America and South America [8, 11]. Moreover, a new cluster of PCV2 has been recently described and proposed as the PCV2e genotype [12].

In South America, PMWS and PCVAD have been reported; phylogenetic studies have only been conducted in Brazil, Argentina, Uruguay and Colombia [10, 13, 14]. The objective of this study is to evaluate the genetic diversity of PCV2 using sequences in pig affected by PMWS obtained from intensive swine farms in Chile.

## Methods

### Samples

The samples used in this study were obtained from fixed tissues submitted between 2005 and 2008 to the Veterinary Pathology Laboratory, University of Concepcion, based on PMWS suspected clinical signs. Samples from 20 intensive pig farms located in 5 administrative regions of Chile Central, plus an extensive wild boar farm were included, and data about geographic localization was registered. Further, clinical PMWS diagnosis was confirmed by macroscopic, microscopic lesions and “in situ” hybridization (ISH) based on the technique described by Segalés and Domingo, 2002 [3]. Subsequently, a subset of 51 PCV2 positive samples were selected for PCR testing and sequencing based on its ISH intensity.

### PCV2 DNA sequencing

Paraffin embedded tissues were manually deparaffinized using xylene. After, the DNA was extracted using QIAamp DNA mini kit (Qiagen, Valencia, USA) following manufacturer’s instructions. First, samples were tested using a selective PCR protocol, which is able to differentiate PCV2a and PCV2b viruses and identify mixed infections [15]. Also, samples were tested with sequencing PCR primers based on protocol described by Quintana et al., 2002 [16]. This PCR protocol amplifies a 652 bp fragment located between genomic positions 940 and 1596. PCR products were visualized in a 1.8% agarose gel and purified using QIAquick PCR Purification Kit. Sequencing was performed directly on the PCR products, which were sequenced on an ABI PRISM 3100 Avant Genetic Analyzer (Applied Biosystems) at Macrogen USA (MD, United States).

### Genotyping study

Molecular characterization of PCV2 strains was performed on the basis of sequence analysis of 462 nt of the ORF2. Sequence alignment was carried out with Clustal W algorithm including reference sequences corresponding to different PCV2 genotypes [8] and sequences from neighbor countries. The degree of identity among nucleotide and amino acid sequences was determined by using BioEdit v. 7.0.5. Phylogenetic tree was reconstructed by the neighbor-joining method using MEGA v5.0 software. Bootstrap values were determined with 1000 replicates of the dataset and PCV1 sequence was included as outgroup (FJ475129).

### Recombination analysis

In order to analyze the potential recombinant strains, an integrated software package, the recombination detection program (RDP v.3.44), was used [17]. The general settings were a highest acceptable *p*-value of 0.01 and Bonferroni correction. A dataset of selected PCV2 sequences of different genotypes (*n* = 18), were included in the RDP analysis excluding known recombinant sequences. Each potential recombinant strain was analyzed separately.

Additionally, SIMPLOT software v. 3.5.1 [18] was employed to further analyze the potential parental PCV2 lineages and putative recombination breakpoints previously analyzed and estimated by RDP software. Four groups of 462 nt of ORF2 sequences were included in SIMPLOT analysis: a group of potential recombinants (O1/2008, M1/2007, L1/2007, H3/2007, H4/2007, B4/2008, C1/2006, B1/2005, B2/2005), a group of a reference sequences from PCV2d genotype (AY943819; JX535296), a group of PCV2a genotype sequences (AY180397; AY146993) and an out-group (PCV1 isolate FJ475129). A similarity plot was generated comparing the percentage of identity versus genome position of the query isolate to the panel of references sequences. Analysis was performed with a window size of 100 nt and a step size of 20 nt. The analysis of potential recombinant strains was also carried out by the identification of conflicting phylogenetic trees in different regions of the alignment based on the putative recombination breakpoints observed in SIMPLOT and RDP analysis. Phylogenetic reconstructions were realized as described above. One phylogenetic tree was constructed on the basis of the minor parent region (nucleotides 100 to 389) and the other comprised the major parent region concatenated alignment of nucleotides 1 to 99 and 390 to 462.

## Results

PCV2 genetic material was amplified by selective PCR in 19 out 51 samples. No mixed infections were identified. Ten samples, tested negative for selective PCR, were amplified by sequencing primers. Therefore, 29 out 51 tissues samples were sequenced, 28 corresponding to 18 intensive swine farms and 1 from an extensive wild boar farm. Genomic sequences were deposited into GenBank™ with accession numbers (KU992866 to KU992894) and each sequence included a farm code and year (Table 1).

**Table 1:** List of PCV2 sequences including farm ID, year of collection, administrative region, and genotype

Farm	Year	Region	Genotype	Sequence ID	Accession No
H	2005	VI	PCV2a	H/2005	KU992879
N	2005	VIII	PCV2b	N/2005	KU992890
B	2005	VIII	PCV2rec	B1/2005	KU992870
B	2005	VIII	PCV2rec	B2/2005	KU992871
K	2006	RM	PCV2b	K/2006	KU992885
N	2006	VIII	PCV2b	N/2006	KU992891
C	2006	VI	PCV2rec	C/2006	KU992875
J	2007	RM	PCV2a	J/2007	KU992874
A	2007	RM	PCV2b	A1/2007	KU992866
A	2007	RM	PCV2b	A2/2007	KU992867
A	2007	RM	PCV2b	A3/2007	KU992868
A	2007	RM	PCV2b	A4/2007	KU992869
B	2007	VIII	PCV2b	B/2007	KU992872
D	2007	VIII	PCV2b	D/2007	KU992876
E	2007	IX	PCV2b	E/2007	KU992877
G	2007	VI	PCV2b	G/2007	KU992892
I	2007	VII	PCV2b	I/2007	KU992884
P	2007	VIII	PCV2b	P/2007	KU992886
R	2007	VIII	PCV2b	R/2007	KU992894
H	2007	VI	PCV2rec	H1/2007	KU992880
H	2007	VI	PCV2rec	H2/2007	KU992887
H	2007	VI	PCV2rec	H3/2007	KU992881
L	2007	RM	PCV2rec	L/2007	KU992888
M	2007	VI	PCV2rec	M/2007	KU992889
F	2008	VII	PCV2b	F1/2008	KU992878
B	2008	VIII	PCV2rec	F2/2008	KU992873
O	2008	RM	PCV2rec	O/2008	KU992893
Q	2013	RM	PCV2a	Q1/2013	KU992882
Q	2013	RM	PCV2a	Q2/2013	KU992883

**Fig. 1:**
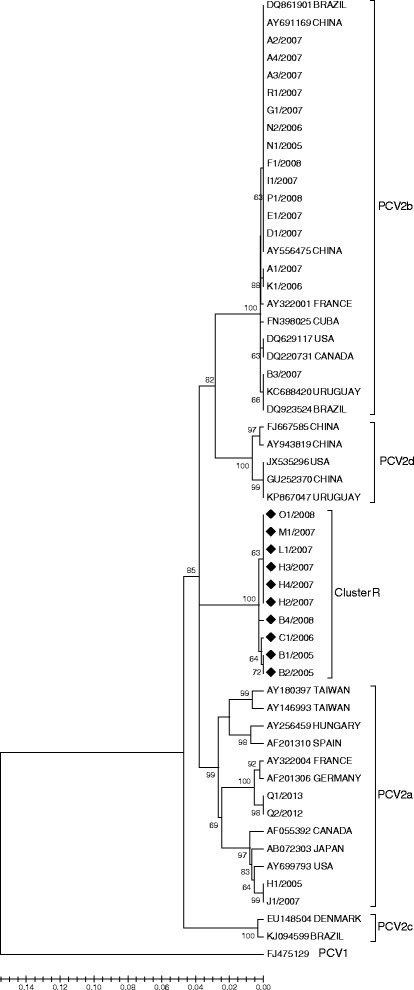
Phylogenetic tree generated by neighbor-joining method using 462-nt sequence of ORF2 PCV2. Chilean strains were compared with twenty-five PCV2 reference strains. PCV1 was included as out-group. GenBank accession numbers and country of origin are specified. Genotypes are indicated with straight brackets

Phylogenetic analysis using the partial ORF2 sequences obtained (462 nt) showed that PCV2 sequences belonged to PCV2b (15 out 29), PCV2a (4 out 29), and 10 sequences segregated independent labeled as Cluster R (Fig. 1). PCV2b Chilean sequences came from 10 pig farms and a nucleotide identity of 99.9% was observed in this group. On the other hand, PCV2a Chilean sequences were obtained from 3 pig farms, sharing a nucleotide identity of 96.9% among them.

**Fig. 2:**
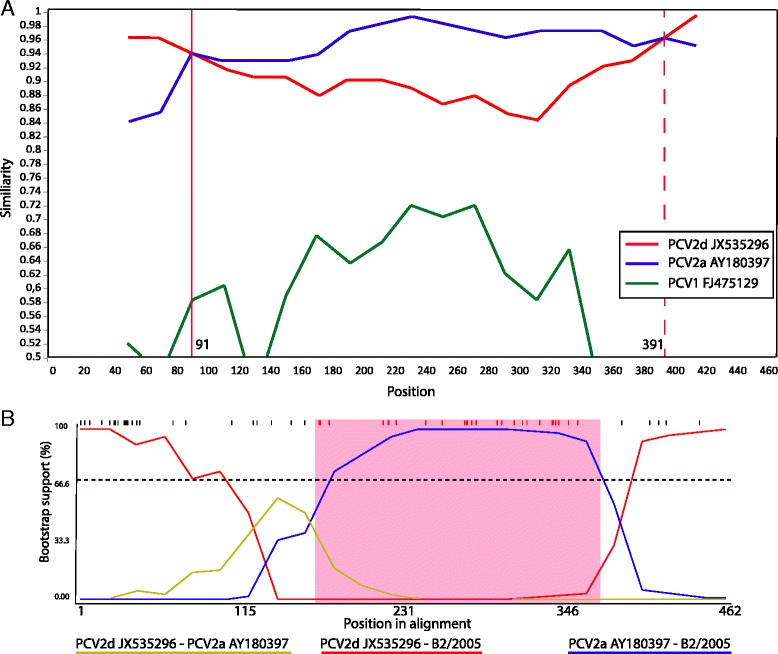
Recombination analysis. **a** Similarity plot generated by SIMPLOT software based on 462 nt of ORF2 sequence using B2/2005 as query sequence and representative parent sequence of genotypes PCV2d (red) and PCV2a (blue) (JX535296 and AY180397, respectively). A PCV1 isolate was used as outgroup (green). The analysis was performed with a window size of 100 nt and a step size of 20 nt. **b** BootScan analysis and potential breakpoints for B2/2005 predicted by RDP3 software

Ten isolates were grouped in a new cluster with a very high statistical support (bootstrap value, 100%) (Fig. 1), suggested the possible presence of recombinant strains. This cluster contained viruses from 6 different pig farms with nucleotide identity of 99.5%. These sequences were detected as recombinants using RDP3 software and the recombination events were statistically supported by BootScan, Maxchi, Chimera and 3Seq methods with average *p*-values of 1.9 × 10^−5^, 6.6 × 10^−4^, 1.6 × 10^−2^ and 4.3 × 10^−3^, respectively. Results showed that all recombinant sequences were originated from the same parental lineages. The parent sequences resulted to be JX535296 (major parent, PCV2d genotype) and AY180397 (minor parent, PCV2a genotype). In addition, a similarity plot analysis was carried out with putative parental sequences using SIMPLOT software to corroborate the results obtained using RDP3 software. In the Fig. 2a is shown the similarity plot generated for B2/2005 sequence as an example, indicated that the recombinant presented a nucleotide similarity with a PCV2a isolate (blue) between nucleotides 91 and 391, and with an PCV2d strain (red) between nucleotides 1 to 91 and 391 to 462. Moreover, recombination graph of the recombination event for B2/2005 is also shown using BootScan method (Fig. 2b) and nucleotides 132 and 386 were predicted by RDP software to be the potential breakpoints.

**Fig. 3:**
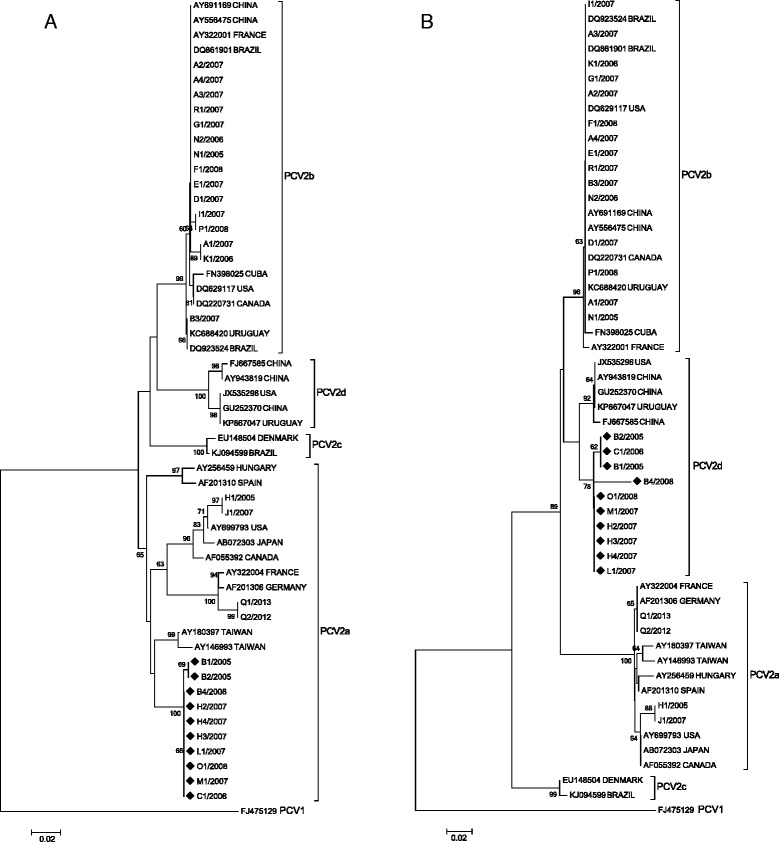
Phylogenetic trees generated by neighbor-joining method based on **a** the minor parent region estimated with SIMPLOT and RDP software (nt 100–389) and **b** the major parent region (concatenated alignment of nt 1–99 and nt 390–462). Chilean strains were compared with twenty-five PCV2 reference strains. GenBank accession numbers and country of origin are specified. Genotypes are indicated with straight brackets. Discordant clustering patterns of potential Chilean recombinant strains with different genotypes are observed (◆)

On the other hand, two phylogenies were constructed based on the major and minor parental regions taking to account data obtained of SIMPLOT and RDP software (Fig. 3). In the phylogeny based on the minor parental region, Chilean potential recombinant sequences clustered within PCV2a genotype (Fig. 3a). However, in the phylogeny based on the major parental region, recombinant viruses resulted to be more closely related to a set of PCV2d sequences (Fig. 3b). The occurrence of incongruent phylogenetic trees strongly provided an additional evidence of recombination. Finally, the closest sequence publicly available presents an identity of 98% (p-distance) and was obtained from Thailand (PCV2/ Thailand/THKUF49/2007, # HQ701666), which correspond a PCV2d virus.

## Discussion

The study conducts the first phylogenetic analysis of PCV2 in Chile. PCV2 isolated from Chilean intensive swine farms, covering over the 90% of the pork production, resulting a complete national perspective of PCV2. The results confirm the high presence of PCV2b (52%) and less represented PCV2a (14%). Surprisingly, 34% of sequences were PCV2a/PCV2d recombinant viruses. The no amplification of these 10 sequences by the selective PCR was further explained due to these primers were not able to amplified PCV2d, the major parenteral of these viruses. Furthermore, we were not able to detect PCV2d variant in the Chilean PCV2 diversity even if was the major parent of the recombinant viruses, probably this event occurred years before and the recombinant remained. PCV2a and PCV2b, and the recombinant viruses were found since 2005. Nevertheless, were not observed geographical, temporal and other patterns, most likely due to there is not enough sequences per farm.

Recently, a large-scale study determinates that the PCV2 vaccination is important factor in the PCV2 ecology, driving the evolution and epidemiology of the virus. PCV2 vaccination by selective pressure generates vaccine scape-mutants, which become new genotypes waves [19]. Interestingly, the vaccination against PCV2 in Chile started in 2007 and massive in swine farms around 2008–2009. This study includes samples since 2005 to 2013, but differences before and after massive vaccination were not reported mostly due low number of isolates per farm and year.

Six farms presented PCV2 recombinant viruses; this event was observed in samples from 2005. However, even if geographical or others patterns were not identified, probably the presence of these recombinant viruses is linked between farms. There are not similar sequences reported with this particular recombination in the world; therefore, probably this recombination event occurred in Chile and then was spread to the rest of the farms.

Recombination events are common among PCV2 strains, which have been reported in several countries [20, 21]. Also, different patterns of recombination have been described, with break points in both ORF1 and ORF2, which suggest that the recombination can occur easily in PCV2 co-infections [22].

On the other hand, PCV2b was the most common genotype in Chilean farms during the PMWS outbreak, as has been found of the rest of the world, where more than 50% of the viral sequences belonged into PCV2b [11]. PCV2b sequences did not show divergence over time among samples obtained in 2005 to 2008 from several different farms and present 99.9% identity with viruses found in the rest of the world.

## Conclusions

This is the first study that revealed the genetic diversity of PCV2 in Chile during a PWMS outbreak. Our findings suggested that a novel cluster of Chilean sequences emerged resulting from recombination between genotypes PCV2a and PCV2d within ORF2. However, analysis of the complete PCV2 genome and further surveillance of PCV2 is needed to confirm the real impact of these recombinant viruses.
